# Phylogenetic and Taxonomic study of a new aquatic alga, *Pleurastrum gracilis* sp. nov. (Volvocales, Chlorophyta), from the Qinghai Lake

**DOI:** 10.3897/phytokeys.279.199681

**Published:** 2026-09-25

**Authors:** Qiufeng Yan, Tao Ge, Degao Wang, Yachen Bai, Benwen Liu, Jifei Hu, Huan Zhu, Guoxiang Liu

**Affiliations:** 1 Anhui Technical College of Industry and Economy, Hefei 230051, China Anhui Technical College of Industry and Economy Hefei China; 2 Anhui Institute of Geological Experiment, Hefei 230001, China Anhui Institute of Geological Experiment Hefei China; 3 Institute of Hydrobiology, Chinese Academy of Sciences, Wuhan 430072, China Institute of Hydrobiology, Chinese Academy of Sciences Wuhan China

**Keywords:** 18S rDNA, ITS2 secondary, new species, phylogenetic analysis, *
Pleurastrum
*, structure

## Abstract

The species of the genus *Pleurastrum* are rarely mentioned, and related research and reports are limited. At present, this genus only contains 8 species, however, there is no record of this genus species in China, and there is a lack of research on its diversity. In this study, we collected two isolates of algae, FACHB-3462 and FACHB-3463, from lakes and freshwater rivers on the Qinghai Tibet Plateau. Based on morphological data and phylogenetic analysis, we described *P. gracilis* in detail and compared its similarity with the morphology of existing species. The phylogenetic tree constructed based on the 18S rDNA sequence indicates that *P. gracilis* is most closely related to *P. amblystomatis*, *P. aquaticum*, and *P. ellipsoideum*, forming a branch independent of the genera *Protosiphon*, *Spongiochloris*, and *Spongioarcinopsis*. The phylogenetic analysis of ITS sequences shows that the isolates also form an independent branch. Different from other species, the isolate was found in a saline lake at a high altitude. The difference between the isolate and its sisters branch was that its cell wall was thin, and it was observed that many ellipsoid cells were connected head to tail, and it had one or more CBCs different from other species in the genus. At the same time, there was a branching structure on Helix-1. Given these morphological differences, phylogenetic analysis results, and comparison of ITS2 secondary structures, the isolate isolated in this study is proposed as a new species and named *Pleurastrum gracilis***sp. nov**.

## Introduction

As a highly ecologically adaptable photosynthetic autotrophic organism, green algae have a wide range of habitats, not only distributed in conventional freshwater bodies, marine ecosystems, and terrestrial habitats, but also able to tenaciously colonize extremely harsh environments such as deserts, alpine snowfields, and polar regions (Sciuto et al. 2023). In recent years, with the widespread application and increasing depth of investigation of molecular phylogenetic techniques, many new evolutionary clades and taxonomic units in the Chlorophyta phylum have been continuously discovered and reported (Prochazkova et al. 2019; Andreoli et al. 1998; Sciuto et al. 2015; Gálvez et al. 2021; Trentin et al. 2022). However, this only reveals the tip of the iceberg of green algae biodiversity, and there is still enormous exploration space for its overall species richness and phylogenetic lineage. This study focuses on the unique ecologically fragile area of the Qinghai Tibet Plateau, where two green algae were successfully isolated from saltwater lakes and freshwater rivers. Given the limitations of traditional morphological identification in the classification of green algae, we adopted a multi gene joint analysis strategy to define the species of the isolates: we selected the nuclear 18S rRNA gene as a conserved molecular marker for higher-order taxa, and combined it with the nuclear core barcode gene – the nuclear transcription spacer (ITS) region, which has higher variation resolution, for sequencing; At the same time, in order to provide more detailed evidence from the perspective of functional and structural evolution, we also conducted in-depth annotation and comparison of the secondary structure of ITS2. Based on the comprehensive molecular evidence presented above, we have classified these two algal isolates as belonging to the genus *Pleurastrum*. Looking back at the classification history of this genus, *Pleurastrum* was first established by scholar Chodat as early as 1894 (Chodat 1894); Despite being over a hundred years old, the species diversity of this genus is still far from sufficient. According to the records of the Algaebase database (Guiry and Guiry 2023), currently this genus only covers 8 valid species worldwide. From the perspective of phylogenetic status, the genus *Pleurastrum* is the only member of the Pleurasraceae family in the Chlorophyta phylum, Chlorophyta class, and Chlorophyta order. The phenomenon of “single genus and single family” and the extremely low species description rate further highlight the research gap that urgently needs to be explored in terms of species diversity and evolutionary mechanisms of this genus.

For a long time, there has been a relative lack of research literature and reports on the genus *Pleurastrum*, which makes it extremely rare and easily overlooked in field sampling and taxonomic identification. However, as a unique group within the Chlorophyta phylum, the genus *Pleurastrum* exhibits strong ecological adaptability and a wide range of habitats. Its species not only inhabit conventional aquatic ecosystems, but are also widely distributed in diverse terrestrial habitats (Chodat 1894; Sciuto et al., 2023). At the morphological level, this genus exhibits extremely high phenotypic plasticity, with its body structure spanning a wide lineage from single-cell spheroids to complex branching filaments (Chodat 1894; Visher 1933; Sciuto et al., 2023). Looking back at the taxonomic history of this genus, since the first establishment of the *Pleurastrum* model species *P. insigne*, many scholars have mainly relied on phenotypic features to define and describe new species within the genus (Visher 1933; Deason and Bold 1960; Tupa 1974; Sluiman and Gärtner 1990). Although early cultivation observations and comparative morphological studies have to some extent expanded our understanding of the species diversity of *Pleurastrum*, relying solely on morphological standards has repeatedly caused confusion and controversy in taxonomy (Deason and Bold 1960; Tupa 1974; Sluiman and Gärtner 1990; Lukešová 1991). This limitation mainly stems from two aspects: on the one hand, some key morphological characteristics of green algae often only appear briefly at specific developmental stages of the cell growth cycle, and due to the extremely short observation window, it is easy to make misjudgments due to omissions; On the other hand, some of the morphological features used as identification criteria are actually convergent or conservative features widely present within the Chlorophyta phylum, lacking sufficient evolutionary specificity and difficult to use as reliable criteria for defining higher-order taxa or distinguishing closely related species. Compared to the limitations of traditional morphology, the phylogenetic analysis that integrates molecular markers with the secondary structure of the internal transcription spacer 2 (ITS2) can provide a more objective and comprehensive perspective on the dual dimensions of genome evolution and RNA structure conservation, thus revealing its hidden species diversity more realistically. In view of this, this study established a multidimensional identification strategy that integrates morphology and molecules: it retains the intuitive comparison of traditional morphological structures and deeply introduces molecular feature analysis based on ITS2 secondary structures; Meanwhile, a phylogenetic tree was constructed by combining multiple gene sequences such as 18SrDNA and ITS. The implementation of this comprehensive research framework aims to break through the bottleneck of traditional classification and provide a solid scientific basis for more accurate analysis and clarification of the species diversity and systematic evolutionary status of the genus *Pleurastrum* in China.

This study aims to combine morphological and molecular phylogenetic analysis of 18S rDNA and ITS sequences, and compare the ITS2 secondary structures of closely related species. Proposed a new species of *Pleurastrum*.

## Materials and мethods

### Isolation, purification, and cultivation of algal isolates

In September 2020, algal isolate FACHB-3463 was collected from the surface of Qinghai Lake in Qinghai Province, China (36°39'N, 100°23'E, altitude: 3198 meters); In April 2021, the same algal species was obtained again in Ganjing River (30°21'N, 108°1'E, elevation: 269 m), Zhongxian County, Chongqing, with the algal isolate number of FACHB-3462. The algal isolate was stored in the freshwater algal culture library of the Institute of Hydrobiology, Chinese Academy of Sciences (located at No. 7, Donghunan Road, Wuhan, Hubei Province). The specific sampling and separation purification methods are as follows: take the water sample and place it in a 2 mL centrifuge tube, dilute it with distilled water, and mix thoroughly with vortex oscillation. Inoculate an appropriate amount of suspension onto BG-11 solid medium containing 1.3% agar, and use a triangular glass coated rod (Allen 1968) for coating. Cultivate the agar plate under constant temperature of 25 °C and alternating light and dark conditions for 12 hours until visible bacterial colonies grow. Select individual algal colonies and transfer them to fresh culture medium for purification until pure culture is obtained. Subsequently, the purified single algae isolate was transferred into a 96 well plate containing BG-11 liquid medium. Using Leica DM5000B microscope(Wetzlar, Germany) to observe the morphology of original and cultured algae isolates, and taking microscopic photos with Leica DFC320 digital camera(Wetzlar, Germany).

### DNA extraction, PCR amplification, sequencing

Total genomic DNA was isolated from pure unialgal cultures after cell lysis using a bead beater (3110BX, Biospec Products, Buttersville, USA) with mini beads, following the protocol of the HP Plant DNA Kit (Omega Bio-Tek, GA, USA). A 50-μL PCR mixture was prepared, comprising 6 μL of DNA template, 1 μL of each primer, and 42 μL of Master Mix. To amplify the 18S rDNA region, primers 18SR and 18SF (Medlin et al. 1988) were utilized under the following thermal profile: an initial denaturation at 94 °C for 5 min; 35 cycles of 94 °C for 50 s, 55 °C for 50 s, and 72 °C for 90 s; and a final extension at 72 °C for 10 min. The ITS region was amplified with primers NS7m and LR1850 (Bhattacharya et al. 1996) using a program of 94 °C for 5 min, followed by 32 cycles of 94 °C for 1 min, 55 °C for 1 min, and 72 °C for 2 min, with a final extension of 72 °C for 10 min. The resulting amplicons were commercially sequenced by TSINGKE Biotechnologies (China) and submitted to the GenBank database (http://www.ncbi.nlm.nih.gov/) under accession numbers: 18S rDNA: PP512554, PP512555; 28S rDNA: PP555647, PP555648; ITS: PP574981, PP574982.

### Molecular phylogenetic analyses

To investigate potential phylogenetic affiliations within the broader green algal lineage, 60 18S rDNA and 25 ITS sequences were retrieved from GenBank based on BLAST query results. These sequences, along with our target sequences, were initially aligned utilizing MAFFT v7.3 (Standley 2013), followed by manual refinement in Seaview v5.0 (Gouy et al. 2021). MEGA v11.0 (Tamura et al. 2021) was employed to assess mutational saturation by plotting pairwise distances against model-corrected distances at variable sites; the results confirmed an absence of saturation for both transitions and transversions. Modeltest-NG (Darriba et al. 2020) was used to identify the optimal evolutionary models via the Akaike information criterion and hierarchical likelihood ratio tests, yielding TN93+G+I for 18S rDNA and K2+G+I for the ITS dataset. Phylogenetic reconstructions were conducted using maximum likelihood (ML) via PhyloSuite v1.2.1 (Zhang et al. 2020) and Bayesian inference (BI) via MrBayes v3.2.2 (Mishra and Thines 2014). For the ML analysis, heuristic searches were executed using 10 random sequence addition replicates coupled with the tree bisection and reconnection (TBR) branch-swapping algorithm, and nodal support was evaluated with 1000 bootstrap replicates. The BI analysis involved four Markov chains (one cold, three heated) running for 20 million generations, sampling trees every 10,000 generations. Convergence was determined when the average standard deviation of split frequencies between parallel runs fell below 0.01. After discarding the initial 25% of sampled trees as burn-in, a consensus topology was generated to calculate posterior probabilities. The final trees were visualized and annotated using Figtree v1.4.3 (http://tree.bio.ed.ac.uk/software/figtree/) .

### Comparison of ITS2 secondary structure

The secondary structure of the ITS2 region was predicted utilizing the ITS2 database (http://its2.bioapps.biozentrum.uni-wuerzburg.de/) to evaluate structural variations among different *Pleurastrum* species. Subsequently, simultaneous alignment of both the ITS2 sequences and their corresponding secondary structures was performed using 4SALE v1.7, based on a ClustalW (Larkin et al. 2007) multiple sequence alignment (Seibel et al. 2008). Finally, the resulting structural models were visualized and refined using Varna v3.1 (Darty et al. 2009).

## Results

### Pleurastrum gracilis

Taxon classificationPlantaeChaetophoralesPleurastraceae

Q.Yan & G.Liu sp. nov.

0B4036FE-C290-54D6-A36E-9C68A635E34E

#### Description.

**Figure 1.**
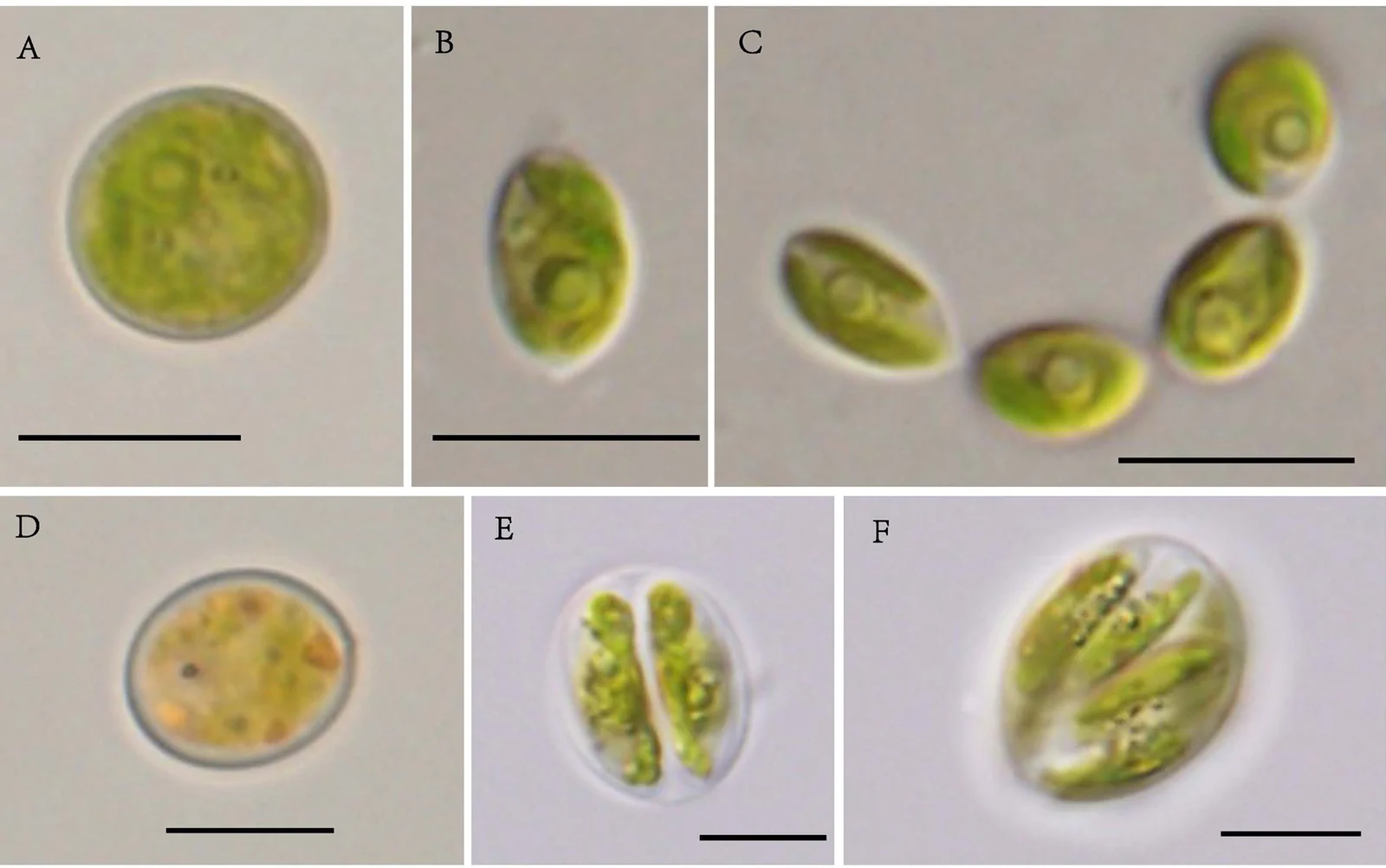
Morphological characteristics of *Pleurastrum gracilis*. **A**. Spherical vegetative cell; **B**. Elliptical cells containing one pyrenoid; **C**. Multiple ellipsoidal cells connected at the head and tail; **D**. Aging cells; **E**. Zoosporangium containing 2 zoospores; **F**. A sporangium containing 4 zoospores. Scale bars: 5 μm.

Single cell, ranging from spherical to ellipsoidal in shape (Fig. 1A, B). The cell wall is thin, the chloroplasts are single, the cell surface is smooth, and there is one pyrenoid (Fig. 1A). Spores are ovate to elliptical in shape. Spherical nutrient cells can have a diameter of up to 11 μm, sporangia can reach 18 μm, and ellipsoidal nutrient cells are 9–12 μm long and 6–9 μm wide. Multiple ellipsoidal cells were observed to be connected (Fig. 1C). The cultured cells often appear yellow green in color (Fig. 1D). Asexual reproduction occurs by producing 8–16 zoospores through sporangia(Fig. 1E, F). No sexual reproduction observed.

#### Etymology.

Species names refer to the morphology of zoospores.

#### Type locality.

Qinghai Lake in Qinghai Province, China (36°39'N, 100°23'E); on the surface of the lake water.

#### Iconotype.

Fig. 1A.

#### Holotype.

QF2021 (HBI), collected by Qiu–Feng Yan, September 2020; deposited in the Freshwater Algal Herbarium (HBI), Institute of Hydrobiology, Chinese Academy of Sciences, Wuhan, Hubei Province, China.

#### Distribution.

At present, the algal isolate is only found in China. It grows on the surface of saltwater lakes and freshwater rivers.

#### Authentic culture.

Culture isolate FACHB-3463 is deposited in the Freshwater Algae Specimen Station, Institute of Hydrobiology, Chinese Academy of Sciences (http://algae.ihb.ac.cn/).

Compared with other *Pleurastrum* species, the isolate was found in a saline lake at high altitude. The difference between the isolate and its sisters branch was that the cell wall was thin, and a number of ellipsoid cells were observed to connect head to tail.

##### Phylogenetic analyses

The 18S rDNA alignment applied 58 sequences (including 8 *Pleurastrum* sequences), which consisted of 1641 sites, out of which 473 (17.8%) and 365 (12.3%) were variable and parsimony-informative sites, respectively. 22 ITS sequences were used for alignment and 361 sites, out of which 171 (59.9%) and 158 (42.5%) were variable and parsimony-informative sites, respectively. Table 1 presents detailed information about the alignment and nucleotide substitution in 18S rDNA and ITS concatenated phylogenies for molecular phylogenetic analysis.

**Table 1.** Detailed information of alignment and nucleotide substitution in 18S rDNA, ITS sequence concatenated phylogenetic for ML analysis.

dataset	18S rDNA	ITS
Alignment length	1641	361
Number of sequence	60	25
Parsimony-information sites	365	158
Invariant sites	1168	190
Best-fit model	TN93+G+I	K2+G+I
Base frequency %(T/C/A/G)	26/21/25/28	23/25/28/25
Saturation test (*Iss/Iss.c*)	0.110<0.836	0.467<0.680

**Figure 2.**
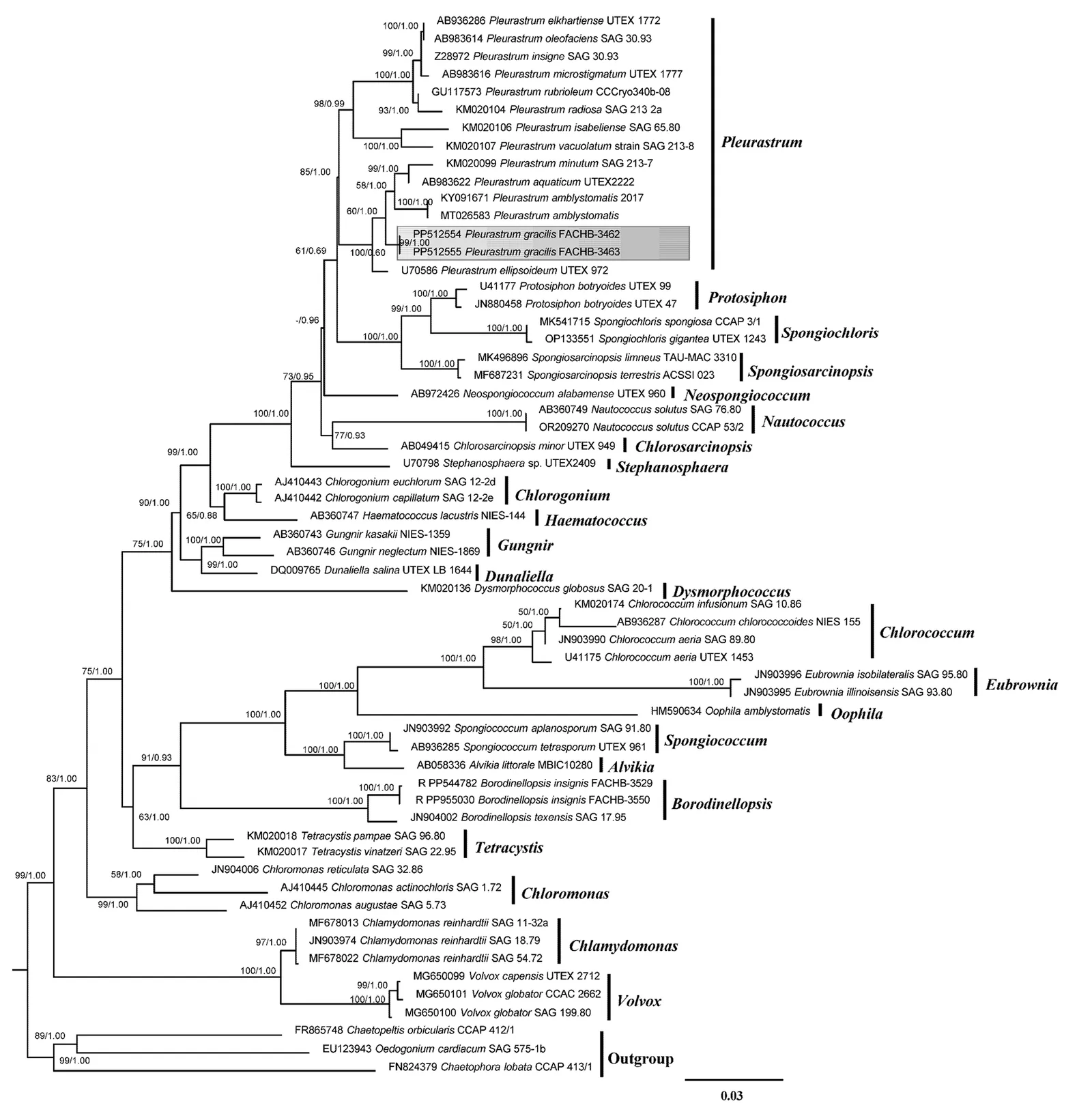
Phylogenetic tree constructed by Bayesian approach based on 18S rDNA sequences. Maximum likelihood bootstrap values and Bayesian posterior probabilities are given on nodes. Values above 0.50 for BI and 50 for ML are given. The gray boxes represent the new species in this study.

**Figure 3.**
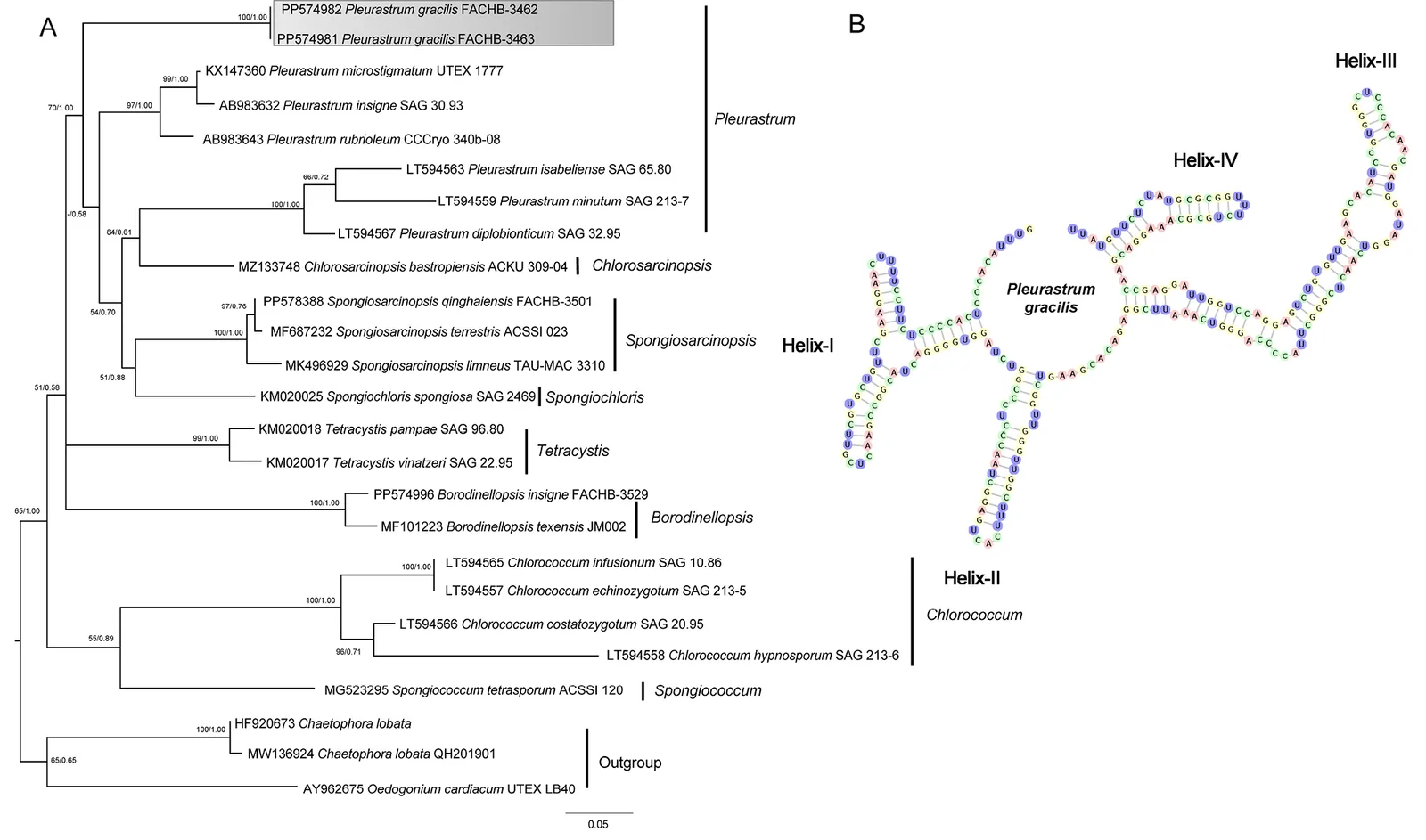
**A**. Phylogenetic tree constructed by Bayesian approach based on ITS sequences. Maximum likelihood bootstrap values and Bayesian posterior probabilities are given on nodes. Values above 0.50 for BI and 50 for ML are given. The gray boxes represent the new species in this study; **B**. ITS2 secondary structure of *Pleurastrum gracilis*.

The phylogenetic tree was constructed using the Bayesian approach based on 18S rDNA and ITS alignments (Figs 2, 3), with the Bayesian posterior probabilities and ML bootstrap support reported. The topology of our 18S rDNA phylogeny (Fig. 2) is essentially consistent with that reported in previous studies (Kim et al. 2025). The tree based on 18S rDNA showed a strong Pleurastrum clade, comprising the algal isolates isolated in the present study; hence, the algal isolates were classified into the genus *Pleurastrum* (Fig. 2). The phylogenetic tree constructed based on ITS sequences supports the above results (Fig. 3A).

##### ITS2 secondary structure

**Figure 4.**
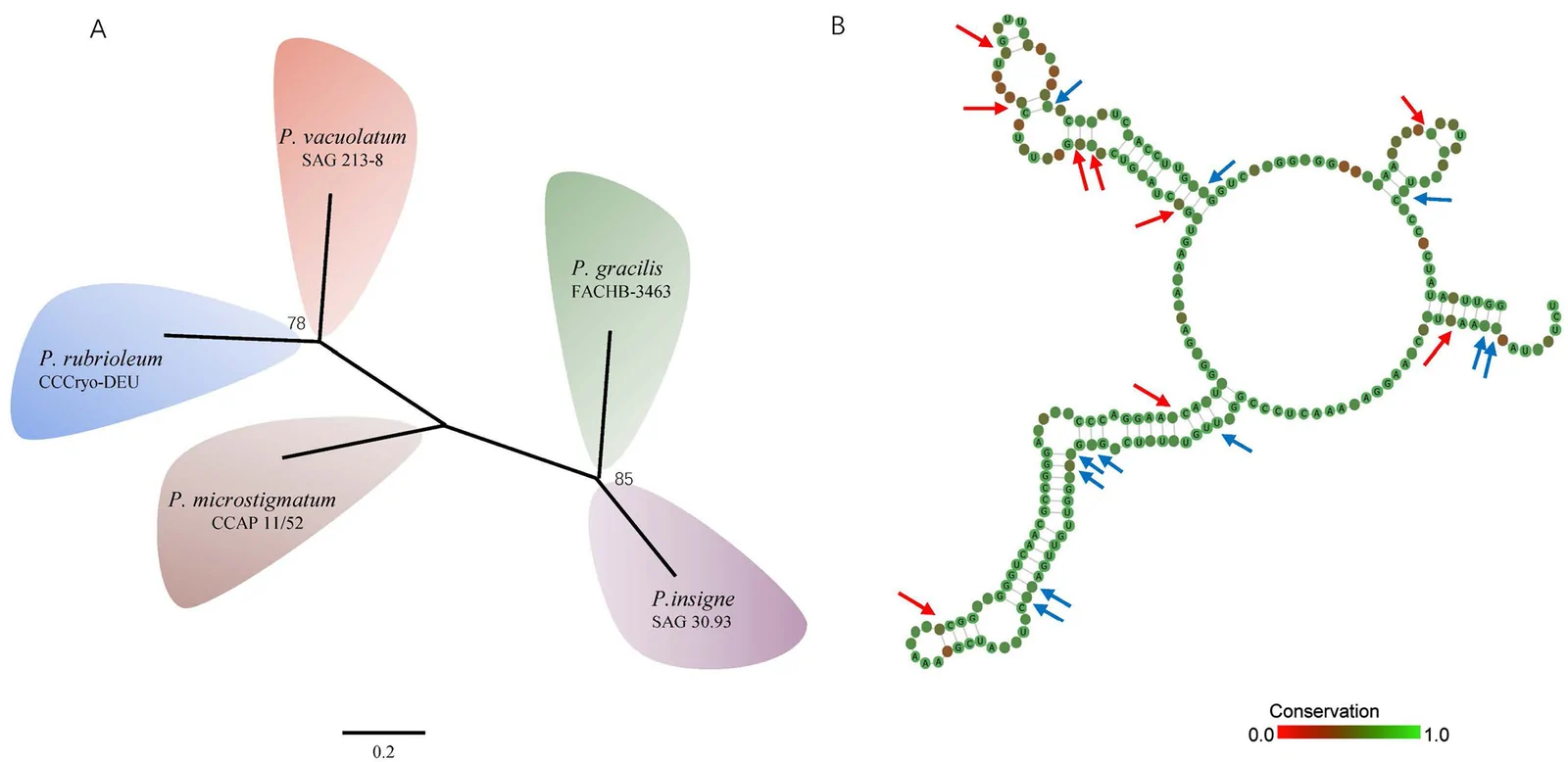
**A**. The phylogenetic tree reconstructed from ITS2 secondary structure and sequence. Maximum likelihood bootstrap values are given on nodes. Different species are circled with different colors as the background; **B**. The ITS2 secondary structure among 5 isolates in *Pleurastrum*. The darker the color, the more significant the variation.

The secondary structure of ITS2 of *P. gracilis* was annotated and predicted (Fig. 3B), and it was found that, similar to the model species, this species has a typical one ring four stem structure of eukaryotes. The differences between this species and known species within the genus were compared, with the only difference being the presence of branching structures on Helix-1. We also constructed a maximum likelihood evolutionary tree using ITS2 secondary structure (Fig. 4A). The shared ITS2 secondary structure was predicted using five species within the *Pleurastrum* genus (Fig. 4B), and it was found that the four main helix regions (Helix) of these two isolates had at least one compensatory base change (CBC) compared to other species within the genus (Table 2).

**Table 2.** CBCs and h-CBCs quantities with 5 *Pleurastrum* sequences.

	***P. insigne***	***P. microstigmatum***	***P. rubrioleum***	***P. vacuolatum***	***P. gracilis***
	**SAG 30.93**	**CCAP 11/52**	**CCCryo-DEU**	**SAG 213-8**	**FACHB-3463**
	**LT594568**	**FR865591**	**HQ404881**	**LT594560**	**PP512555**
*P. insigne* SAG 30.93 LT594568	*/*	3	1	4	1
*P. microstigmatum* CCAP 11/52 FR865591	3	*/*	4	3	1
*P. rubrioleum* CCCryo-DEU HQ404881	1	4	*/*	4	1
*P. vacuolatum* SAG 213-8 LT594560	4	3	4	*/*	1
*P. gracilis* FACHB-3463 PP512555	1	1	1	1	*/*

## Discussion

The genus *Pleurastrum* was established by Chodat in 1894. Although new species have been continuously reported in recent years (Sciuto et al. 2023; Kim et al. 2025), according to AlgaeBase records, this genus currently only contains 8 species. As a monotypic genus of the Pleurasraceae family, its species diversity urgently needs to be further explored; Throughout the entire Chlorophyta phylum, even with the emergence of new evolutionary clades and species (Andreoli et al. 1998; Sciuto et al. 2015; Prochazkova et al. 2019; Gálvez et al. 2021; Trentin et al. 2022), Its overall biodiversity is still far from fully revealed. Looking back at the taxonomic history of this genus, since the description of the *Pleurastrum* type species *P. insigne*, species definition has long relied on morphological features (Visher 1933), but a single phenotype criterion can easily lead to taxonomic confusion (Deason and Bold 1960). The introduction of molecular markers and phylogenetic analysis of ITS2 secondary structure can effectively compensate for this deficiency. Therefore, this study adopts a strategy that combines morphology and molecules, based on multi gene sequences such as nuclear SSU rDNA and ITS, supplemented by ITS2 secondary structural features, to more accurately analyze the species diversity of the *Pleurastrum* genus in China.

In the phylogenetic analysis, a Bayesian tree constructed based on 18S rDNA showed that multiple *Pleurastrum* species, including the isolated isolate in this study, clustered into a highly supported monophyletic branch, which was significantly distinguished from genera such as *Protosiphon*, *Spongiochloris*, and *Spongioarcinopsis* (Fig. 2). Therefore, these isolates were classified under the genus Pleurastrum. The phylogenetic tree constructed based on ITS sequences also confirmed this attribution (Fig. 3A), and showed high ML support and Bayesian posterior probability at the intra species, inter species, and closely related genus levels. At the intra genus level, *P. gracilis* has formed an independent branch: although it is most closely related to *P. amblystomatis*, *P. aquaticum*, and *P. ellipsoideum*, they have clear boundaries with each other. The ITS Bayesian tree also supports the phylogenetic independence of *P. gracilis* (Fig. 3A). In addition, ITS2 secondary structure analysis showed that the four main helix regions (Helix) of these two isolates had at least one compensatory base change (CBC) with other species within the genus, and had a unique branching structure on Helix-1. Given that the emergence of CBC is often regarded as an important indicator for defining species or genus level taxonomic units (Vanormelingen et al. 2007; Bock et al. 2011a, b), the differences in the above secondary structures further confirm the independent species status of *P. gracilis*.

In terms of morphology, *P. gracilis* exhibits significant differences: its cell wall is thinner than that of *P. insigne* and *P. microstigmatum*, which have different wall thicknesses; At the same time, this species exhibits the phenomenon of multiple ellipsoidal cells connected end-to-end, which has not been reported in other species within the genus. In terms of ecological habits, species of the genus *Pleurastrum* are widely distributed in aquatic and terrestrial ecosystems (Chodat 1894; Sciuto et al. 2023), while the isolates in this study were collected from both low altitude freshwater rivers and high-altitude saline lakes. This indicates that the species has a broad ecological range and is tolerant to extreme high-altitude environments. Previous studies have shown that green algae can thrive in high-altitude extreme habitats (Andersen 1992; Malcata et al. 2018), and this characteristic of *P. gracilis* endows it with potential ecological and economic research value.

## Conclusions

Based on phylogenetic analysis, morphological features, and ITS2 secondary structure comparison, *P. gracilis* is proposed as a new *Pleurastrum*. As the only genus within its family, this genus covers a limited number of species, and there should be increasing research on the diversity of this genus and its family.

## Supplementary Material

XML Treatment for Pleurastrum gracilis

## Additional information

### Artificial Intelligence (AI) use

The authors accept full responsibility for the content of the manuscript, including the disclosure of any use of AI.

No AI tools were used in the preparation of this manuscript.

### Author ORCIDs

Q. Yan https://orcid.org/0000-0002-6362-6081

### Data availability

All of the data that support the findings of this study are available in the main text.
